# A rare primary hydatid cyst of the psoas muscle in a rural setting: A case presentation

**DOI:** 10.1016/j.amsu.2020.09.002

**Published:** 2020-09-09

**Authors:** Yassine Merad, Hichem Derrar, Ahmed Zeggai, Malika Belkacemi, Zoubir Belmokhtar, Haiet Adjmi-Hamoudi

**Affiliations:** a Department of Parasitology-Mycology, Central Laboratory, « Hassani Abdelkader » Hospital, University Center, Sidi-Bel Abbès, Djellali Liabes University, Faculty of Medicine, Sidi-Bel Abbès, Algeria; b Laboratory of Environmental Information Synthesis, Djillali Liabes University, Sidi-bel-Abbes, Algeria; c Department of Pulmonary Medicine & Lung Diseases, « Hassani Abdelkader » Hospital, University Center, Sidi-Bel Abbès, Djellali Liabes University, Faculty of Medicine, Sidi-Bel Abbès, Algeria; d Department of Surgery, “Ben Badis” Hospital, Sidi-Bel-Abbès, Algeria; e Department of Hemobiology and Blood Transfusion, « Hassani Abdelkader » Hospital, University Center, Sidi-Bel Abbès, Djellali Liabes University, Faculty of Medicine, Sidi-Bel Abbès, Algeria; f Department of Biology and Environment, University of Science and Biotechnology, Oran, Algeria; g Department of Parasitology-Mycology, University of Algiers, Algeria

## Abstract

Hydatid cyst is a zoonosis caused mainly by the larval stage of the cestode worm *Echinococcus granulosus,* hydatidosis is frequently found in sheep-raising countries such as the Mediterranean countries. The disease usually involves the liver (75%) and lung (15%). We describe a case of hydatid cyst of the psoas muscle; we are reporting this case because of its rarity and its difficulty to diagnose clinically.

A 16 –year-old female from rural setting, presented to the department of surgery of our institution, with complaints of right flank pain. On abdominal examination, there was sensitization in the right lower quadrant, and there was no resistance or rebound.

On abdominal ultrasonography, a 6 × 6 cm hydatid cyst was detected within the psoas muscle, which was confirmed by positive indirect hemagglutination, no other organ involvement has been detected by CT scan. A pericystectomy was performed; the intact intramuscular cyst was completely excised, Macroscopic and microscopic examination of the specimen confirmed Hydatid cyst. The patient was discharged from hospital on the fifth postoperative day. No local recurrence was detected during postoperative follow up.

Hydatidosis should be considered in cases of a symptomatic swelling in musculoskeletal system without history of trauma and irradiation when patients belong to endemic area.

Cyst or complex retroperitoneal tumors, cold or pyogenic abscess of psoas muscle are considered in differential diagnosis.

In the light of this case and the literature data, we discuss the diagnosis and the therapeutic problems raised by hydatid cyst of the psoas muscle.

## Highlights

• Hydatid cyst is a parasitic common disease in sheep breeding areas, especially in Mediterranean countries.
• Hydatid cyst of the psoas muscle is important to know because of its rarity and its difficulty to diagnose clinically, especially in young people.
• Hydatid cyst should be considered in the presence of any painless cyst mass of muscle in endemic regions. Unilateral and primitive forms are mainly reported.
• Excision of the intact intramuscular cyst completely is required to avoid contamination and recurrence.
• Cyst or complex retroperitoneal tumors, cold or pyogenic abscess of psoas muscle are considered in differential diagnosis.

## 1 Introduction

Hydatid Disease (HD) is a zoonosis caused mainly by the larval stage of the cestode worm *Echinococcus granulosus* or dog tapeworm. Dog is the primary host in echinococcal infestation while the intermediate hosts are sheep, cattle, horses, and occasionally man. For this reason, HD has its highest incidence in sheep and cattle-rearing regions, such as the Mediterranean countries. The human can incorporate to the parasitic life cycle as the intermediate host, role that is generally played by sheep. Organs affected by HD are the Liver (63%), Lungs (25%), Muscle (5%), Bones (3%), Kidney (2%), Brain (1%), and spleen (1%). Primary hydatidosis of skeletal muscle is therefore rare, with reported prevalence of 0.5–5% in the endemic areas [1,2].

The psoas muscle is an uncommon location for HD and accounting for only 1–3% of cases [2], not less than 70 cases of psoas hydatid cysts have been cited [3].

The selectivity of the proximal muscles would be due to the importance of the blood flow [4].

HD of psoas muscle occurs during childhood and involves young adults [1,5,6], and can manifest as painless mass of muscle. The clinical symptoms of atypical hydatid cysts range from non-specific complaints to anaphylaxis and death. The clinical manifestation of the disease is formed by localization and pressure effect of the slowly growing cyst in the infected organ.

These are the following possible complications of psoas muscle hydatid cyst: surinfection and fever [7], femoral nerve injury [8], ureteral compression [9], psoas hydatid cyst revealed by inguina hernia [10], non-functional kidney by compression [11]. This work has been reported in line with the SCARE criteria [12].

## 2 Case

A 16 –year-old female from rural setting, presented to the department of surgery of our institution, with complaints of right flank pain, few episodes of vomiting from last 3 days.

There was no motor deficit in both lower extremities. On abdominal examination, there was sensitization in the right lower quadrant, and there was no resistance or rebound. No urinary and genital troubles were reported, and there was no pressure symptom except a growing mass.

On abdominal ultrasonography, a 6 × 6 cm unilocular swelling arising from right psoas muscle, and was suggestive of hydatid cyst”

**Fig. 1:**
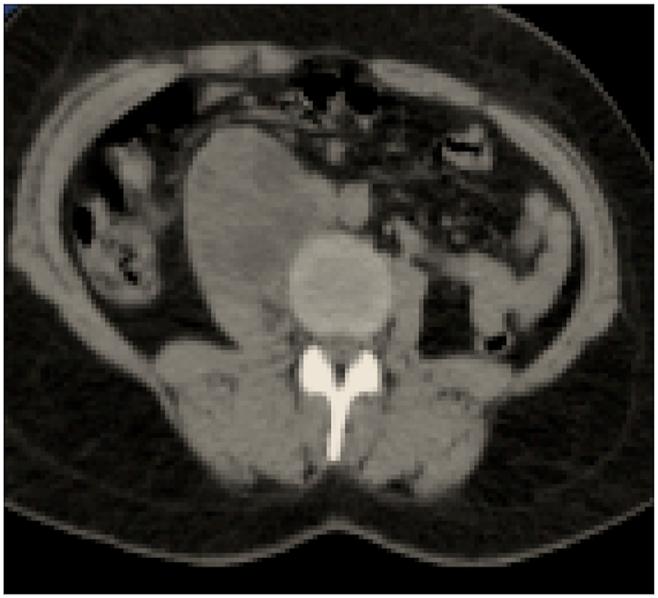
Abdomino pelvic scan showing the presence of a collection in the psoas muscle and the right ilio psoas.

We performed whole-body scanning for hydatid cyst via abdominal–thoracic–cranial computed tomography (CT), because of the of the patient, and the endemic which detected no hydatid cysts in other organs or tissues (Fig. 1). Radiographics findings were compatibles with hydatid cystic mass within psoas muscles associated to slight calcifications within the cyst wall, which was confirmed by positive indirect hemagglutination (HAI) routine test (titer>1/160).

**Fig. 2:**
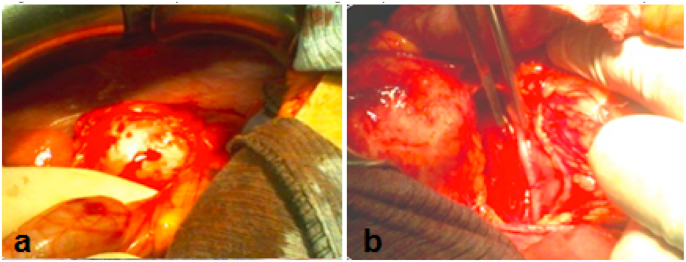
(a,b) psoas hydatid cyst surgical removal.

**Fig. 3:**
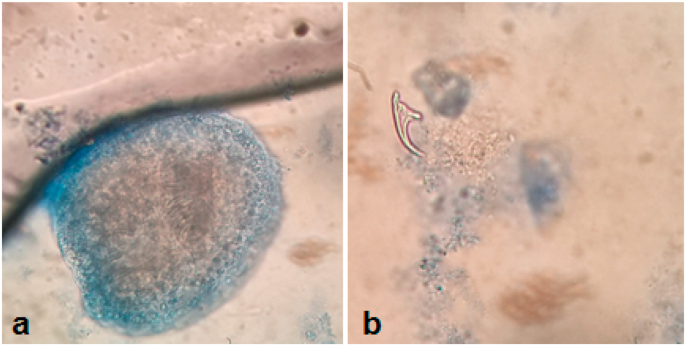
Postoperative microscopic diagnosis of Hydatid cyst: protoscolese (a), hooklet (b).

The patient was prepared for surgery (blood tests, bath), Exploratory done by midline incision, after a protection by fields soaked in the hydrogen peroxide and the inhalation of the content of the cyst and cleaning by serum and hydrogen peroxide. A pericystectomy was performed, the intact intramuscular cyst was completely excised, in order to prevent contamination and recurrence (Fig. 2) microscopic examination is described (Fig. 3).

The patient was discharged from hospital on the fifth postoperative day with a prescription for albendazole (2 × 400 mg/day) for three months.

No local clinical recurrence was detected during postoperative follow up period within one year.

## 3 Discussion

The hydatidosis is a disease of the rural world in the traditional breeding areas. It is an anthropozoonosis due to the development of Man's larva of the *taenia Echinococcus granulosis*. Embryonic eggs, eliminated from the exterior environment with the saddles of the dog, are ingested, penetrate into the digestive wall, through the liver, sometimes exceed the hepatic veins and reach the lungs [13].

The muscular HD is a rare affection and the effect of the psoas seems exceptional; even in country of endemic disease [14], HD of psoas muscle occurs during childhood and involves young adults [1,5,6] from rural setting [4], which concord with our findings. 66 cases reports of hydatid cyst of psoas are listed in pubmed.

The muscle is generally very resistant in the hydatidosis because it tends to divide and encapsulate the larva as well as the contractile activity and the production of lactic acid [13,15].

Some authors claim that muscular HD is due to direct implantation through a wound (e.g. dog bite), but most authors believe larva can reach the muscles from the systemic circulation after leaving the intestine and passing through two filters: the liver and the lungs [15].

Clinical features of hydatid cyst varies from the presence of a swelling or mass to pressure symptoms, cyst infection and rarely anaphylactic shock [[1], [16]] [Mellis], cyst or complex retroperitoneal tumors, cold or pyogenic abscess of psoas muscle are considered in differential diagnosis [17].

Psoas muscle HD may cause ureteric compression and hydronephrosis [9], in our patient there was no pressure symptom except a growing mass. Unilateral and primitive forms are mainly reported [1].

Ultrasonography (US) is particularly useful for the detection of cystic membranes, septa, and hydatid sand as well as the staging and follow-up of the cysts. Computed tomography (CT) best demonstrates cyst wall calcification and cyst infection as well as the passage of contents through a defect [18].

CT scan facilitate diagnostic and therapeutic procedures in order to ensure complete surgical resection [19].

Muscular hydatidosis should be always considered in patients with previous history of hydatid cyst [20], the research of a primary hydatid cyst should be performed by CT scan, abdominal-thoracic computed tomography is sufficient in asymptomatic brain patient and does not require whole-body scanning, but aberrant localisations are frequent in our country, the patient is young and the hydatid evolution is long.

HAI, enzyme-linked immunosorbent assay, or Western blott are carried out for diagnosis, screening and postoperative follow up for recurrence; but these tests are often negative or inadequate for definitive diagnosis if the cyst is intact, calcified or sterile [5].

After performing the pericystectomy and completely intramuscular cyst excision, posoperative prescription of Albendazole is not justified, Muscular hydatidosis should always be considered as secondary localization of the disease, especially in cases of previous surgical dissemination [20], post-operative administration of Albendazole was justified in this endemic area, and for this young patient.

Partial pericystectomy and cyst drainage seems to be the best approach due to its minimal invasiveness. An alternative to surgery is the US-guided cyst puncture and injection of protoscolicide [20].

We emphasise that HD should be taken into consideration in the differential diagnosis of a cystic painless mass in every anatomical location, especially when appearing in young patient from an endemic area.

## Declaration of competing interest

All authors declares that they have no conflict of interest.

## Sources of funding

This case report is not funded by a specific project grant, authors received no financial support for the research, authorship, and publication of this article.

## Ethical approval

Patient gave consentement for photos and publication, in accordance with ethical committee of our Hospital.

Written informed consent was obtained from participant.

Consent from legally authorized representatives parents.

Personal details of patient in any part of the paper or supplementary materials were removed before submission.

## Consent

Written informed consent was obtained from the patient for publication of this case report and accompanying images. A copy of the written consent is available for review by the Editor-in-Chief of this journal on request.

## Author contribution

Study concept and design Yassine Merad, Ahmed Zeggai.

Writing paper: Yassine Merad, Hichem Derrar.

Data collection Ahmed Zeggai, Yassine Merad.

Manuscript review Zoubir Belmokhtar, Haiet Adjmi-Hamoudi.

## Registration of research studies

1. Name of the registry:
2. Unique Identifying number or registration ID:
3. Hyperlink to your specific registration (must be publicly accessible and will be checked):

## Guarantor

Yassine Merad, I am the principal author and I accept full responsibility for the work and the conduct of the study, and I control the decision to publish with agreement of co-authors.

## Provenance and peer review

Not commissioned, externally peer reviewed.
